# Retraction

**Published:** 1855-10

**Authors:** 


*Retraction.*—In our editorial department of last month we published a lachrymose obituary of our friend Prof. Charles Ap A. Bowen. A few days since this lamented individual, of whom we had honestly supposed that

> "His bones lay bleaching on the coral rocks,
> Full many a fathom down,"

(Query—*would* bones bleach in such a situation?) protruded his phiz inside our sanctum door. He was evidently no ghost—his cheeks stuck out with fatness—he was a gross carnality!

A gentleman inquiring for lead-colored gloves at a fashionable mourning store, was requested by the officious clerk to "*step into the mitigated affliction department!*" It is needless to say that, in this instance, we followed the advice of the clerk.

A profuse hæmoptysis in London, seems to have given origin to the report of our friend's death. We have threatened him—in revenge for his untimely reäppearance—with an entire retraction of *everything* we said about him, but having finally concluded that what is written is written, we merely "back down" on the statement of the death, assuring our readers, however, that if not true just now, it, in all probability, will be true sometime during the present century.

